# Multi-Strategy Improvement of Coal Gangue Recognition Method of YOLOv11

**DOI:** 10.3390/s25071983

**Published:** 2025-03-22

**Authors:** Hongjing Tao, Lei Zhang, Zhipeng Sun, Xinchao Cui, Weixun Yi

**Affiliations:** School of Coal Engineering, Shanxi Datong University, Datong 037000, China; 220857002141@sxdtdx.edu.cn (H.T.); zhipengsun@sxdtdx.edu.cn (Z.S.); 220857002125@sxdtdx.edu.cn (X.C.); 230857002147@sxdtdx.edu.cn (W.Y.)

**Keywords:** YOLOv11n, BiFPN, coal gangue recognition, object detection

## Abstract

The current methods for detecting coal gangue face several challenges, including low detection accuracy, a high probability of missed detections, and inadequate real-time performance. These issues stem from the complexities associated with diverse industrial environments and mining conditions, such as the mixing of coal gangue and insufficient illumination within coal mines. A detection model, referred to as EBD-YOLO, is proposed based on YOLOv11n. First, the C3k2-EMA module is integrated with the EMA attention mechanism within the C3k2 module of the backbone network, thereby enhancing the model’s feature extraction capabilities. Second, the introduction of the BiFPN module reduces computational complexity while enriching both semantic information and detail within the model. Finally, the incorporation of the DyHead detector head further enhances the model’s ability to express features in complex environments. The experimental results indicate that the precision (P) and recall (R) of the EBD-YOLO model are 88.7% and 83.9%, respectively, while the mean average precision (mAP@0.5) is 91.7%. These metrics represent increases of 3.4%, 3.7%, and 3.9% compared to those of the original model, respectively. Additionally, the frames per second (FPS) improved by 10.01%. Compared to the mainstream YOLO target detection algorithms, the EBD-YOLO detection model achieves the highest mAP@0.5 while maintaining superior detection speed. It exhibits a slight increase in computational load, despite an almost unchanged number of parameters, and demonstrates the best overall detection performance. The EBD-YOLO detection model effectively addresses the challenges of missed detections, false detections, and real-time detection in the complex environment of coal mines.

## 1. Introduction

As one of the most significant energy sources in China, the country’s coal output is projected to reach 4.71 × 10^10^ tons by the end of 2023 [1]. This figure is expected to account for approximately 66.6% of the total primary energy production for that year. During the coal mining and preparation processes, more than 10% of gangue waste is generated [2]. The combustion of this gangue within coal not only diminishes the actual calorific value but also releases a substantial amount of toxic gases, resulting in severe environmental pollution. Consequently, the accurate, rapid, and effective separation of coal gangue is essential for enhancing coal quality and promoting sustainable development in coal mining operations.

The conventional methods for separating coal from gangue primarily include manual sorting, selective crushing, heavy medium separation, wind separation, jigging coal preparation, and other techniques. Traditional manual sorting is characterized by high labor intensity and low efficiency. In contrast, the heavy medium method and selective crushing techniques often face challenges such as high equipment costs, significant environmental pollution, and operational complexity [3,4,5]. With advancements in artificial intelligence technology, intelligent coal preparation has increasingly become a primary focus of research in this field. Common methods for identifying coal and gangue include ray recognition, infrared thermal imaging, visual recognition, and multispectral recognition, among others [6].

Traditional image recognition requires the manual configuration and extraction of image features through artificial methods. Chen Li et al. [7] utilized wavelet techniques to denoise coal gangue images and developed wavelet moments for feature extraction. They then differentiated between coal and gangue based on the extracted feature values, thereby improving the efficiency of coal separation from gangue. Shen Ning et al. [8] utilized support vector machines and the Relief algorithm to extract 28 features from images of coal and gangue. They then screened these features to develop an optimal classifier for recognition purposes. This method improved the accuracy of identifying raw coal with diverse appearances. Cheng Gang et al. [9] capitalized on the differences in heat absorption capacity between coal and gangue by capturing their images with an infrared thermal imager. They subsequently employed support vector machines to classify and identify these images, thereby demonstrating the feasibility of infrared thermal imaging for coal and gangue recognition. Yang Huigang et al. [10] were the first to determine the thickness of coal and gangue by scanning images captured with a CCD camera. They then employed X-ray imaging for identification, which reduced the impact of thickness on the visual representation of coal and gangue. Li Hequn et al. [11] proposed an Otsu threshold segmentation method based on the Gaussian pyramid to obtain images of coal and gangue. They employed a gray area size matrix and a gray level co-occurrence matrix for feature extraction, subsequently utilizing a support vector machine for classification and recognition to mitigate the impact of lighting variations on the images. Yu Le et al. [12] distinguished between coal and gangue by partially compressing the gray levels of the images while simultaneously analyzing four characteristic parameters derived from the gray level co-occurrence matrix. This approach offers a novel technical method for the separation of coal and gangue.

The image recognition method based on deep learning has gained widespread application in the field of coal and gangue identification due to its high accuracy, rapid detection speed, robust performance, and other beneficial characteristics. Shan Pengfei et al. [13] proposed an algorithm that employs an enhanced Faster R-CNN framework to tackle the problem of excessive dust generated during fully mechanized top coal caving mining. They developed a test bench to validate the identification and localization of coal gangue at the moment of caving. Du Jingyi et al. [14] introduced an enhanced SSD algorithm that incorporates GhostNet as a replacement for the backbone network of SSD. This modification results in a lightweight network structure that effectively detects small targets associated with coal and gangue. Wang Deyong et al. [15] proposed an enhanced algorithm based on YOLOv5s, which achieved efficient detection in complex environments characterized by high noise levels and low illumination. Guo Yongcun et al. [16] introduced a method to optimize the convolutional neural network (CNN) algorithm by implementing weight migration and simplifying the neuron model. This approach enhances the performance of small target detection while preserving spatial information. Teng Wenxiang et al. [17] developed a coal gangue recognition algorithm based on the HGTC-YOLOv8n model, which significantly improved the recognition capability for overlapping and occluded targets.

Guanghui Xue et al. [18] proposed a lightweight YOLO coal gangue detection algorithm based on ResNet18, which significantly enhanced both the real-time performance and accuracy of the model’s detection capabilities. This improvement was achieved by substituting the backbone network and implementing feature-scale clipping in conjunction with unstructured pruning. Honggaung Pan et al. [19] proposed an enhanced YOLOv3-tiny algorithm for the detection of coal gangue. This approach incorporates the spatial pyramid pooling (SPP) network, the squeeze-and-excitation (SE) module, and dilated convolution techniques to achieve rapid and efficient sorting of coal gangue while simultaneously reducing computational complexity. Deyong Shang et al. [20] proposed a lightweight coal gangue recognition algorithm that enhances YOLOv5s by incorporating the SimAM attention mechanism and the GhostNet backbone network. This approach aims to achieve a balance between model efficiency, computational load, and parameter count, particularly under low-light conditions.

With the advancement of intelligent construction in coal mining, there has been a growing emphasis on the intelligent sorting of coal gangue. Although significant progress has been made in current detection methods for coal gangue sorting, these methods still demonstrate limitations in balancing real-time processing, detection accuracy, model deployment, and the detection of small targets. Although the YOLOv3 algorithm demonstrates high precision, it has limitations in real-time performance and detection efficiency. In contrast, although the YOLOv5 algorithm has made significant progress in enhancing real-time performance, its robustness is still insufficient for the detection of targets in complex backgrounds. As a newly released algorithm, YOLOv11’s performance does not yet fully meet the requirements for coal and gangue sorting tasks, particularly in challenging environments, thereby presenting ongoing challenges.

In response to the complex challenges posed by mixed coal waste and motion ambiguity during the detection of coal gangue, this study introduces the EBD-YOLO algorithm for the identification of coal and gangue. This approach aims to address the limitations inherent in existing methodologies. This approach aims to overcome the limitations inherent in existing methodologies. Specifically, an EMA attention mechanism [21] has been integrated into the C3k2 module, leading to the development of the C3K2-EMA module. This new module replaces all existing C3k2 modules within the backbone architecture, thereby enhancing the surface feature extraction capabilities of the coal and gangue model. The bidirectional feature fusion module, BiFPN, is integrated into the Neck layer to more effectively combine feature information from coal and gangue images and enhance the efficiency of model processing. By replacing the original model’s target detection head with DyHead [22], which integrates a self-attention mechanism, the model can significantly enhance its focus on critical feature areas after multiple downsampling processes. This modification improves the representation capability, spatial perception, and feature expression of the detection layer, thereby enhancing the model’s performance in detecting small targets. Through these innovations, the proposed method not only effectively addresses the limitations of existing approaches in adaptability to complex environments, and real-time detection capabilities, but also enhances the model’s accuracy and efficiency. Furthermore, it is better equipped to tackle the challenges associated with intelligent sorting tasks in coal mines.

## 2. Method and Principle

### 2.1. YOLOv11 Model

YOLOv11 is the latest target detection algorithm released by Ultralytics [23]. Its primary network architecture comprises three components: the backbone, neck, and head. The YOLOv11 model is an enhancement developed by Ultralytics based on the YOLOv8 framework. A significant innovation of this model is the replacement of all C2f modules in YOLOv8 with C3k2 modules. Additionally, it introduces the C2PSA module, which follows the SPPF in the backbone, applies the head theory from YOLOv10 within its classification detector [24], and incorporates two depthwise separable convolutions.

The C3k2 module is replaced by the standard C2f module when the parameter C3k is set to false. Conversely, when the parameter C3k is set to true, the Bottleneck module of C3k3 is replaced with the C3 module. This modification enhances the model’s capacity to capture important features while significantly reducing computational requirements. The C2PSA module is inspired by the PSA module of YOLOv10 and builds upon the C2f module. By leveraging the C2 mechanism in conjunction with a stacked PSA module, it significantly enhances the model’s feature extraction capabilities through the integration of a squeeze-and-excitation (SE) attention mechanism. The concept of depthwise separable convolution is introduced following the head module of YOLOv10. Although the detection speed is slightly slower than that of the YOLOv8 detector head, there is a substantial reduction in both the computational load and the number of parameters within the model.

### 2.2. EBD-YOLO Network Structure

**Figure 1:**
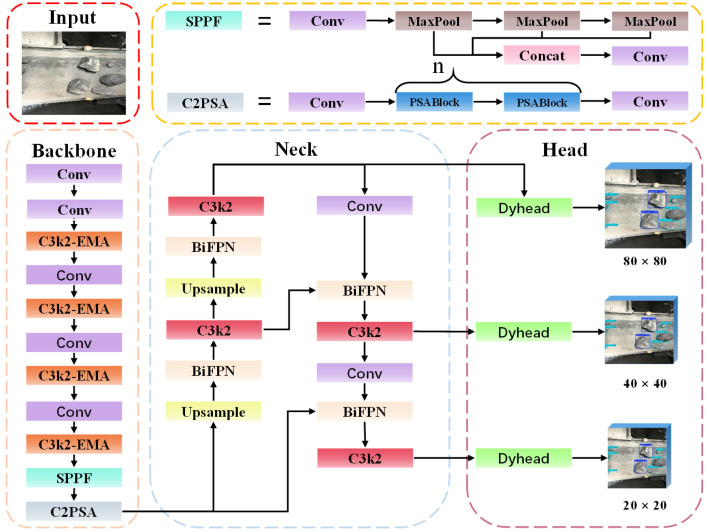
EBD-YOLO network structure.

In light of the challenges associated with missed detections, false detections, and low detection accuracy of coal and gangue under the complex working conditions prevalent in sorting operations, traditional image recognition methods encounter significant limitations. These factors include high costs associated with application equipment and extended relative detection times. To improve detection accuracy and generalization capability, we utilize an enhanced EBD-YOLO model for detection purposes, considering the balance between model deployment and performance. The network architecture of the EBD-YOLO model is illustrated in Figure 1 below.

#### 2.2.1. Improve C3k2 Module

**Figure 2:**
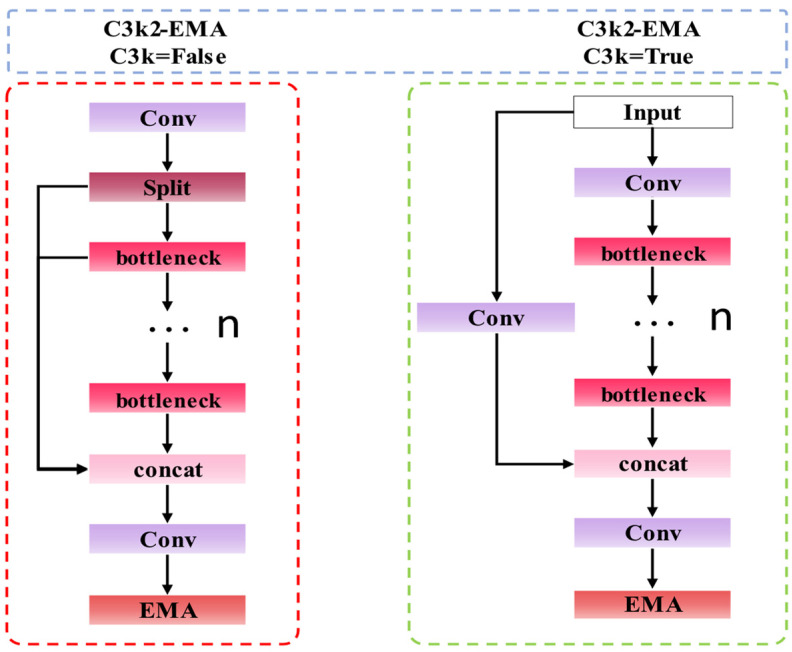
C3k2-EMA structure.

In YOLOv11, the C3k2 module is utilized for feature extraction. When distinguishing between coal and gangue, variations in shape, size, and texture characteristics of these materials can lead to differences in image quality. This disparity ultimately contributes to improved model performance. The introduction of the EMA attention mechanism into the C3k2 module results in the creation of the C3k2-EMA module, which significantly enhances the multi-scale feature processing capabilities of the network model. The structural diagram is presented in Figure 2.

**Figure 3:**
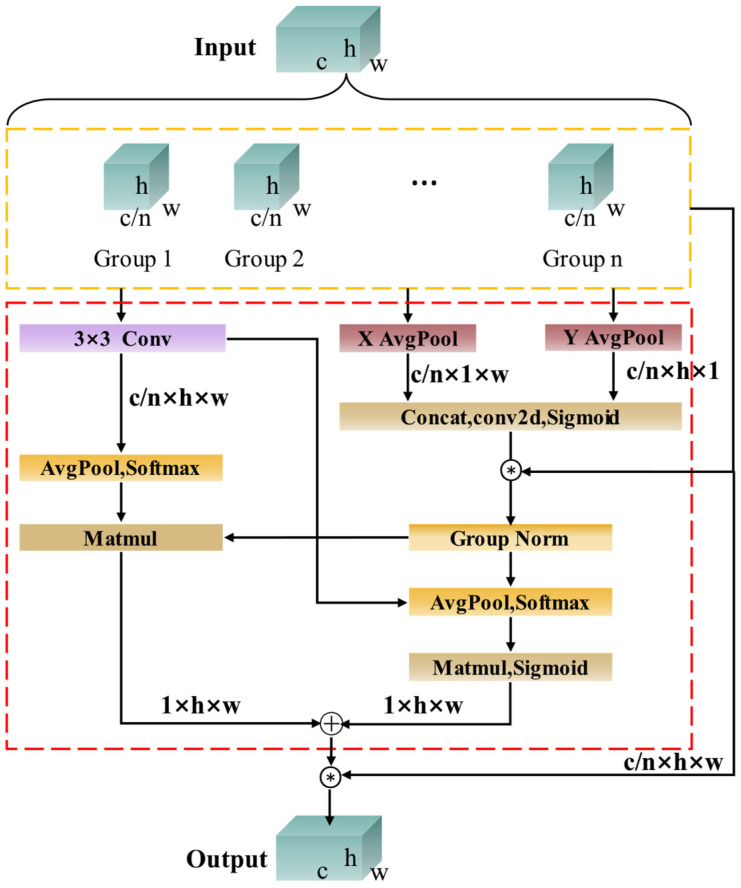
EMA attention mechanism structure.

The attention mechanism is commonly utilized to identify and process essential information in object detection tasks. This approach can significantly improve the model’s sensitivity to coal and gangue targets, thereby enabling the extraction of more comprehensive feature information. Consequently, the EMA attention mechanism is introduced to enhance the information fusion capability between channels through feature grouping, parallel sub-networks, and cross-spatial learning methods. By preserving channel information, specific channel dimensions are converted into batch dimensions to enable cross-dimensional interaction within sub-networks. Ultimately, the outputs of these parallel networks are combined to enhance the information fusion capability among channels. The structural diagram of the EMA attention mechanism is illustrated in Figure 3.

The EMA attention mechanism enhances the CA attention mechanism by decomposing the channel into two one-dimensional feature encodings. Furthermore, it includes a parallel branch that employs a convolutional kernel size of 3 × 3.

For the given feature *X* ∈ *R^c×h×w^*, group feature grouping is utilized to partition the feature dimension into n sub-features. In the case of a 1 × 1 convolution branch, the input feature map undergoes one-dimensional global average pooling across the two spatial dimensions, followed by concatenation, activation, and multiplication operations facilitate the exchange of information across dimensions. For the 3 × 3 branch, we extract additional multi-scale information from the input feature map using a 3 × 3 convolution. Subsequently, cross-spatial learning is employed to perform two-dimensional average pooling and maximum pooling operations on the 3 × 3 branch. This process yields dimensional representations of $R_{3}^{c/n\times 1\times 1}$ and $R_{3}^{c/n\times h\times w}$, respectively.

The feature channel output encoded by the 1 × 1 branch after passing through group normalization is denoted as $R_{1}^{c/n\times h\times w}$, while the unified feature dimension is represented as $R_{1}^{c/n\times h\times w}{\times R}_{3}^{c/n\times 1\times 1}$; Conversely, the feature channel output encoded by the 1 × 1 branch following group normalization can be expressed as $R_{1}^{c/n\times 1\times 1}$, and the unified feature dimension after aggregation is given by $R_{1}^{c/n\times 1\times 1}\times R_{3}^{c/n\times h\times w}$.

Finally, the matrix resulting from the multiplication is utilized to combine the output feature information from each group, thereby producing a set of two spatial attention weight values. After applying the sigmoid activation function, the final output feature map is generated, ensuring that spatial information is preserved without increasing the model’s complexity. The formula for the two-dimensional global pooling operation is presented in Equation (1):

(1) $$Z_{c}=\frac{1}{h\times w}\sum_{j}^{h}\sum_{i}^{w}x_{c}\left(i,j\right)$$

#### 2.2.2. BiFPN Module

**Figure 4:**
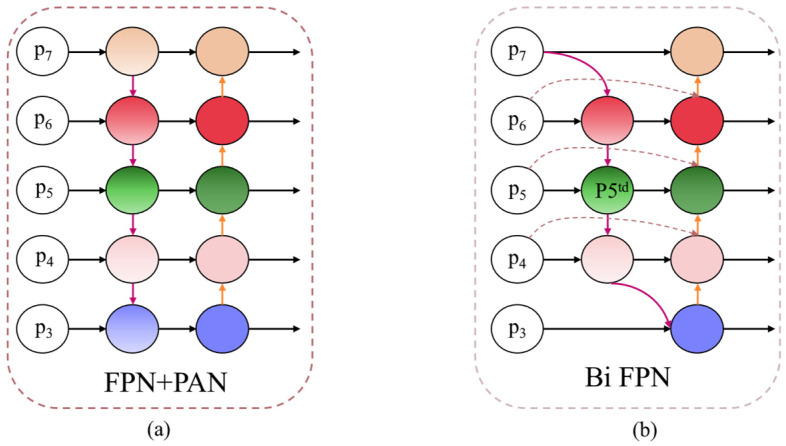
Feature fusion network structure ((**a**) FPN + PAN; (**b**) BiFPN).

As a conventional feature fusion network, the feature pyramid network (FPN) [25] aggregates multi-scale features in a top-down manner, enhancing the transmission of high-level semantic information throughout the feature fusion process. In contrast, the path aggregation network (PANet) utilizes a bottom-up approach for feature fusion, effectively transmitting low-level detailed information to the upper layers [26]. The structural designs of FPN and PANet are illustrated in Figure 4a. YOLOv11 incorporates an FPN + PAN configuration as its feature fusion network within the neck network, thereby combining both approaches to ensure that the final feature map retains both high-level semantic information and low-level detail information.

In addressing the challenges of low resolution and limited feature information in certain small target images related to coal and gangue within this research task, we employed BiFPN as a substitute for the PANet network. This approach enabled effective feature extraction from small target images. The structure of the BiFPN feature network is illustrated in Figure 4b. Compared to PANet, BiFPN employs a strategy that integrates bidirectional cross-scale connections and weighted characteristics to eliminate nodes that contribute minimally to the overall features of the network. Additionally, the introduction of an additional channel between the original input and output nodes allows the network to integrate more information without incurring extra computational costs. Finally, each bidirectional path is considered a feature network layer involved in the feature fusion process, thereby expanding the target receptive field and acquiring more comprehensive semantic information.

BiFPN specifically utilizes fast normalization weighted feature fusion during the feature integration process, effectively partitioning the input information from various features to achieve a balanced representation of that information. The underlying principle is illustrated in Equation (2):

(2) $$O=\sum_{i}\frac{\omega_{i}I_{i}}{\varepsilon +\sum_{j}\omega_{j}}$$

Among them, *O* denotes the output feature map, *I_i_* signifies the input feature map, and *ω_i_* and *ω*_j_ represent the learning weights of the network. The experimental results indicate that the fused BiFPN effectively enhances connectivity among different levels of the feature pyramid within the YOLOv11 network, thereby improving its detection capabilities for coal and gangue across various scales.

#### 2.2.3. Improve the Detection Head

**Figure 5:**
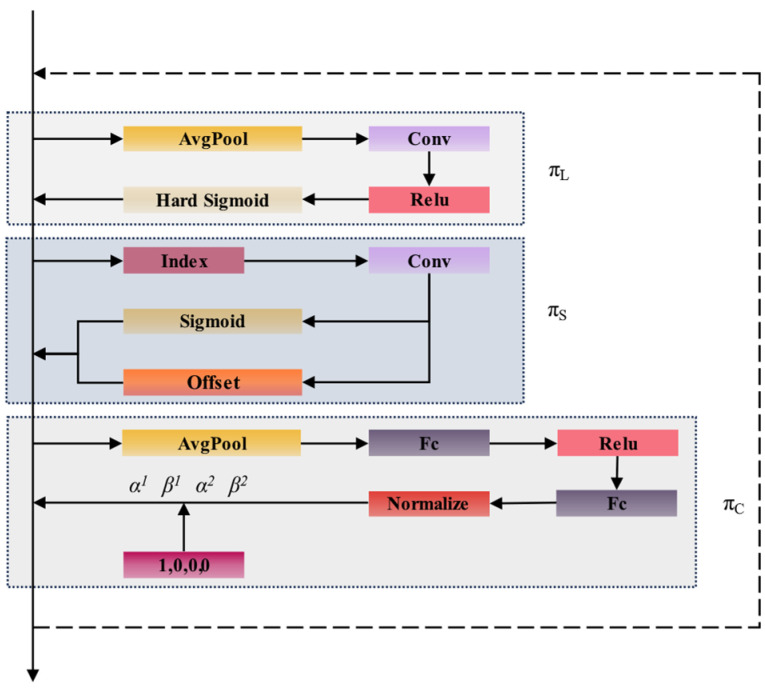
DyHead network structure.

DyHead (dynamic detection head) is a detection head used in object detection tasks that integrates attention mechanisms to process input feature information across multiple levels (L), spatial dimensions (S), and channels (C). It achieves this through unified scale perception, spatial perception, and task perception. By dynamically adjusting the size of the receptive field, DyHead enhances the model’s robustness, enabling it to manage complex scenes more effectively. Additionally, it facilitates the multi-scale fusion of feature information, thereby improving the model’s detection capabilities for images with varying pixel resolutions. The structural diagram of DyHead is illustrated in Figure 5.

For a given three-dimensional feature *F* ∈ *R*^*L*×*S*×*C*^, the feature map utilizes three sequential attention mechanisms: the scale attention mechanism (*π_L_*), the spatial attention mechanism (*π_S_*), and the task attention mechanism (*π_C_*). The resulting calculation formula for the attention function is presented in Equation (3):

(3) $$W\left(F\right)=\pi_{C}\left(\pi_{S}\left(\pi_{L}(F)\cdot F\right)\cdot F\right)\cdot F$$

The formulas for the scale attention mechanism, spatial attention mechanism, and task attention mechanism are presented in Equations (4), (5) and (6), respectively:

(4) $$\pi_{L}(F)\cdot F=\sigma \left(f\left(\frac{1}{SC}\sum_{SC}F\right)\right)\cdot F$$

(5) $$\pi_{S}\left(F\right)\cdot F=\frac{1}{L}\sum_{l=1}^{L}\sum_{k=1}^{K}\omega_{l,k}\cdot F\left(l;p_{k}+△p_{k};c\right)\cdot \Delta m_{k}$$

(6) $$\begin{array}{l} \pi_{c}\left(F\right)=\max\left(\alpha^{1}\left(F\right)\cdot F_{C}+\beta^{1}\left(F\right)\right),\\ \alpha^{2}\left(F\right)\cdot F_{C}+\beta^{2}\left(F\right) \end{array}$$

where *σ* denotes the activation function; *f* represents the transformation function; *p_k_* + Δ*p_k_* signifies the displacement offset; Δ*m_k_* refers to an additional weight; *K* indicates the number of sparse sampling positions; *F_C_* corresponds to the feature map on the C-th channel; *α* is defined as the attention weight coefficient; and *β* serves as a weighted coordination. Compared to traditional detection heads, DyHead utilizes attention mechanisms within the detection head to enable multi-scale processing of feature map information. This approach significantly enhances the model’s feature representation capabilities and improves its accuracy in detecting coal and gangue targets, thereby contributing to the real-time detection of these materials.

## 3. Experiment and Analysis

### 3.1. Experimental Environment Configuration

The computer hardware configuration used in this experiment is a Windows 10 operating system, Inter (R) Core (TM) i5-12400F CPU @ 2.5 GHz processor, RTX 4060Ti graphics card, 32 GB running memory, and software environment configuration is Python 3.10.14 + PyTorch 2.2.2 + CUDA12.1. The model parameters are set as follows: input image size of 640 × 640, training cycle of 300 rounds, batch size of 16, initial learning rate of 0.01, momentum parameter of 0.937, weight decay factor of 0.0005, and 50 early stopping rounds.

### 3.2. Experimental Dataset

**Figure 6:**
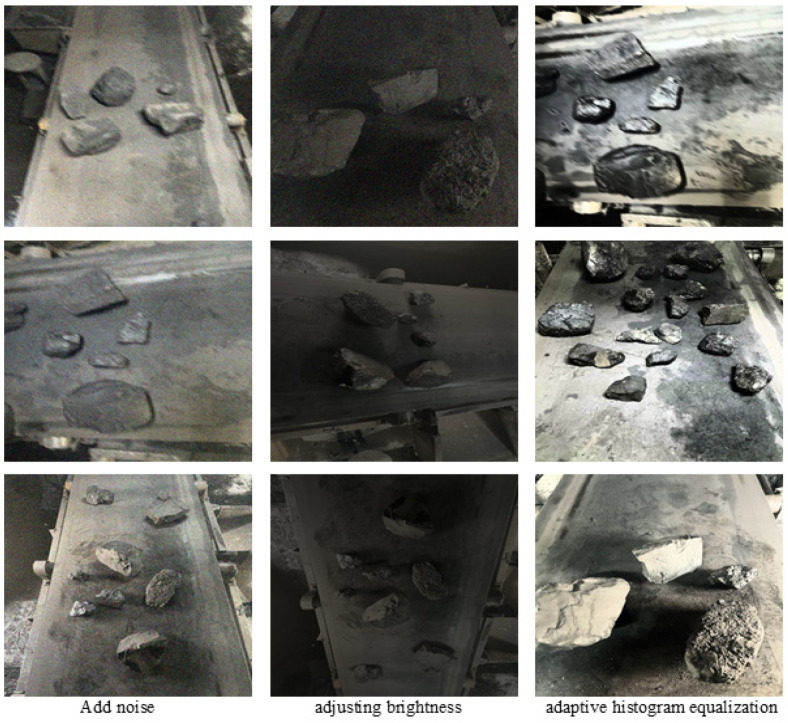
Some data enhancement methods.

The dataset used in this study is a self-constructed collection consisting of coal and gangue sourced from a coal preparation plant in Datong City, Shanxi Province. The video footage was captured using the ZHS2580 intrinsically safe explosion-proof camera (better, Shenzhen, CA, CA), the resolution of the acquired image is 1920 × 1080. Subsequently, Potplayer software, version 1.7.21953, was utilized to periodically extract frames from the video, resulting in the selection of 495 valid images. To address the challenges posed by dust, noise, occlusion, and other issues encountered during the coal gangue sorting process, we expanded the dataset to 2000 samples using various data augmentation techniques. These techniques included flipping, adaptive histogram equalization, adding noise, and adjusting brightness. This expansion aims to enhance the model’s generalization capabilities, improve its robustness, and mitigate the risk of overfitting during the training phase. In the data annotation phase, LabelImg was utilized to annotate the images, assigning labels for coal and gangue accordingly. Ultimately, the dataset, which comprised 2000 images, was divided into a training set, a validation set, and a test set in a ratio of 7:2:1. The resulting distributions of images were 1400 for the training set, 400 for the validation set, and 200 for the test set. A partial dataset image of data enhancement is shown in Figure 6.

### 3.3. Model Evaluation Indicators

To verify the performance of the improved model, experiments were conducted using precision (P), recall (R), and mean of average precision (mAP@0.5). As the main evaluation criteria for model accuracy detection, floating point operations per second (FLOPS), parameters, and frames per second (FPS) are also used to measure model performance. The calculation formulas for precision P and recall R are shown in Formulas (7) and (8):

(7) $$P=\frac{TP}{TP+FP}$$

(8) $$R=\frac{TP}{TP+FN}$$

Among these metrics, true positive (TP) denotes the number of instances that the model correctly identifies; false positive (FP) indicates the number of incorrect identifications made by the model; and false negative (FN) represents the number of actual positive samples that the model misclassifies as negative. The formula for calculating mean average precision (mAP) can be derived from precision (P) and recall (R), as illustrated in Equation (9):

(9) $$\begin{array}{l} AP=\int_{0}^{1}P\left(R\right)dR,\\ mAP=\frac{1}{n}\sum_{i=1}^{n}AP_{i} \end{array}$$

Among these metrics, *AP* denotes the recognition accuracy for a single category, while *n* represents the type of image. Generally, the mean average precision (mAP) is calculated at an intersection over union (IoU) threshold of 0.5, which reflects the average detection accuracy across all categories. A higher mAP value indicates superior model performance.

The FLOPS (floating point operations per second) of a model quantifies the number of floating-point operations executed in one second. Parameters serve as a metric for evaluating the size and complexity of the model. A model characterized by a high parameter count indicates a greater consumption of storage and computational resources, while a model with fewer parameters is generally more lightweight.

### 3.4. Ablation Experiment

To evaluate the optimization effects of each module presented in this article, ablation experiments were conducted using the YOLOv11n benchmark model. The results of these experiments are summarized in Table 1.

Table 1 demonstrates that model ① replaces the C3k2 component in the original model with the C3k2-EMA module, resulting in a 0.7% increase in mAP@0.5 for the model. This finding suggests that the incorporation of the C3k2-EMA effectively addresses the information loss issue encountered during the feature extraction process inherent to traditional C3k2 models.

By incorporating an attention mechanism into the C3k2 module, the model’s ability to extract multi-scale features is significantly enhanced. Model ② introduced BiFPN independently within the original framework, resulting in a 1.5% increase in the *p* value and a 1.2% improvement in mAP@0.5. Furthermore, this modification achieved the greatest reduction in model parameters among all modules.

This suggests that the BiFPN module can achieve both a lightweight design and enhanced performance while also improving the detection of similar features between coal and gangue. Model ③ incorporates the DyHead module independently, resulting in a 0.3% improvement in mAP@0.5 and a 4.3% increase in R, albeit with a slight rise in the number of parameters and computational complexity. This indicates that the DyHead module significantly enhances the model’s detection capabilities for coal and gangue by integrating three attention mechanisms into a unified framework.

In Model ④, the C3k2-EMA module and the BiFPN module are integrated into the original architecture. This modification results in an increase of 1.5% in mAP@0.5 and 83.4% in R. Although the number of parameters is slightly reduced, the FPS is decreased by 0.2%. The findings indicate that the C3k2-EMA module and the BiFPN module mutually enhance one another. Together, these two modules significantly improve detection outcomes by augmenting feature extraction capabilities and facilitating multi-scale feature fusion, although this comes at the expense of some FPS.

Model ⑤ represents the original framework that integrates both the C3k2-EMA module and the Dyhead module. This model demonstrates an increase in mAP@0.5 by 3.3%, with P and R improving by 3% and 4.4%, respectively. Additionally, FPS are enhanced by 5.3%. The results indicate that integrating the C3k2-EMA module with the Dyhead module significantly enhances detection performance. Furthermore, although the model’s parameter count increases by 21.7%, this increment is still lower than that observed in other models.

Model ⑥ incorporates the BiFPN module, building upon model ③. This integration results in a 1.5% increase in mAP@0.5, along with improvements of 2.3% in both precision and recall and an enhancement of 8.2% in FPS. This demonstrates that model ⑥ effectively combines the bidirectional feature fusion capabilities of BiFPN with the computational efficiency of Dyhead, thereby improving detection accuracy and FPS.

In summary, the enhanced model ⑦ exhibits the highest overall performance. It accomplishes this by maintaining high efficacy while only marginally increasing the number of parameters. Additionally, it provides superior capabilities in real-time detection, effectively addressing the practical requirements for identifying coal and gangue in complex backgrounds.

### 3.5. Contrast Experiment

#### 3.5.1. Comparative Analysis of Different Models

To validate the advantages of the enhanced model proposed in this study for coal and gangue detection, experiments were conducted under conditions identical to those used for current mainstream object detection algorithms, including YOLOv3-tiny, YOLOv5s, YOLOv8n, YOLOv10n, and YOLOv11s. The results are presented in Table 2. Given the constraints of the hardware detection platform, it is essential to minimize both the parameter count and computational complexity of the selected model to maintain high detection accuracy.

According to Table 2, the highest values for P, R, and mAP@0.5 were 88.7%, 83.9%, and 91.7%, respectively. In comparison to the baseline model YOLOv11n, the values for R and mAP@0.5 are 3.4%, 3.7%, and 3.9% higher, respectively. Although the computational and parameter requirements of the enhanced model have increased, there has been a 10.01% improvement in FPS.

Compared to the baseline model YOLOv11s, the P, R, and mAP@0.5 exhibited increases of 0.6%, 1.6%, and 0.9%, respectively. Although the improved model requires a higher computational load and an increased number of parameters, it has achieved a significant enhancement in FPS by 10.01%.

Compared to YOLOv11n, which demonstrates higher detection accuracy, the improved model shows increases in P, R, and mAP@0.5 of 3.4%, 3.7%, and 3.9%, respectively. Furthermore, the model’s parameter count and computational complexity have been significantly reduced, effectively addressing practical detection requirements.

In comparison to the YOLOv3-tiny and YOLOv5s models, mAP@0.5 increased by 5.2% and 1.9%, respectively. Additionally, the model’s parameter count decreased by 70.0% and 62.9%, respectively, while computational complexity was reduced by 38.8% and 50%, respectively. Compared to the EBD-YOLO model, the FPS of the YOLOv3-tiny and YOLOv5s models increased by 4.8% and 3.8%, respectively. While these two models exhibit enhanced real-time performance relative to the EBD-YOLO model, they contain a significant number of parameters and FLOPS, which may impede their practical deployment in real-world applications.

Compared to the lightweight YOLOv8n model, the improved model exhibits a slight increase in computational complexity and reduces FPS by 9.1%. However, it achieves a 3.1% increase in mean average precision at a threshold of 0.5 (mAP@0.5). In comparison to the lightweight YOLOv10n, the enhanced model shows a decrease in FPS of 16.4%. While the YOLOv10n model achieves the highest FPS, the enhanced version effectively reduces both computational and parameter complexity while maintaining high performance. Notably, it demonstrates a 4.7% improvement in mAP@0.5, thereby achieving a better balance between detection accuracy.

**Figure 7:**
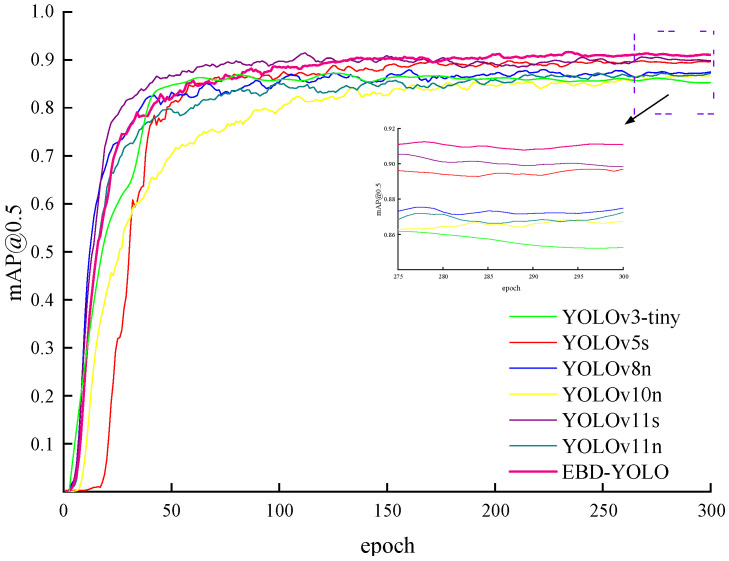
The mean average precision curves for various models.

To further illustrate the detection performance of the enhanced model, various models were evaluated using mAP@0.5, as depicted in Figure 7. In summary, the improved model proposed in this study effectively balances performance metrics when compared to other mainstream models. It demonstrates commendable results in both detection accuracy and computational efficiency, surpassing most conventional algorithms. Consequently, it is well suited for the complex environments encountered at coal gangue sorting sites.

#### 3.5.2. Comparison of Different C3k2 Modules

To evaluate the effectiveness of the C3k2-EMA module, comparative experiments were conducted by replacing the benchmark model YOLOv11n with the C3k2-CA, C3k2-ECA, and C3k2-Faster modules under identical experimental conditions. The results of these experiments are presented in Table 3 below.

As illustrated in Table 3, the parameters and FLOPS of all module types remain largely consistent. Among these modules, the C3k2-EMA module demonstrates the highest mAP@0.5, achieving a value of 88.5%. However, it is important to note that the FPS for this module has decreased by 2.4%. The mAP@0.5 of the C3k2-CA module has decreased by 0.2%, while the model’s FPS has increased by 1%, reaching a value of 83.5 f·s^−1^, albeit with a slight compromise in detection accuracy. In contrast, the mAP@0.5 of the C3k2-ECA module has improved by 0.3%; however, the FPS of this model remains largely unchanged. The C3k2-Faster module achieves the highest FPS with an increase of 2.0%, although this improvement comes at the cost of some reduction in detection accuracy. The C3k2-CA and C3k2-ECA modules provide an impressive balance, while the C3k2-Faster module effectively meets the requirements for both a lightweight design and real-time performance. Furthermore, the C3k2-EMA module greatly improves detection accuracy, achieving an optimal balance among detection precision, computational load, and frame rate. This configuration successfully addresses the dual demands of accuracy and real-time processing in practical applications. In the YOLOv11n model, the primary function of the C3k2 module is feature extraction. In summary, the C3k2-EMA module, which demonstrates superior detection accuracy, has been selected for subsequent experiments. This module offers significant advantages and effectively meets the requirements for multi-scale feature extraction, as well as the specific demands associated with coal rod stone sorting.

#### 3.5.3. Supplementary Verification

To verify the superiority of the improved algorithm compared to other YOLO models, experiments were conducted using algorithms from relevant research literature. These experiments employed the dataset presented in this paper under identical experimental parameters, resulting in the findings summarized in Table 4. As illustrated in Table 4, the EBD-YOLO algorithm exhibits a 4.1% increase in mAP@0.5 compared to HGTC-YOLOv8n. While the model’s parameters and FLOPS remain largely unchanged, there is a significant improvement of 30.3% in FPS. The EBD-YOLO algorithm, in comparison to the SG-YOLO algorithm, shows an increase in mAP@0.5 by 3.9%, a reduction in parameters by 29.3%, and an improvement in FPS by 13.3%. Compared to the SS-YOLOv3-tiny and EBD-YOLO algorithms, the mAP@0.5 has increased by 1.2%. Furthermore, there has been a significant reduction in both the number of parameters and FLOPS, while FPS has improved by 94%. According to the data presented, utilizing YOLOv3 as the benchmark model can significantly improve the system’s detection accuracy. However, at this stage, both the real-time performance and detection efficiency of the model are still suboptimal. Compared to the YOLOv5 and YOLOv8 models within the same series, the YOLOv11 model serves as the benchmark for performance evaluation. This enables the final enhanced algorithm, EBD-YOLO, to strike a balance between real-time processing capabilities and detection accuracy while addressing practical deployment challenges.

### 3.6. Visual Analysis and Experiments

**Figure 8:**
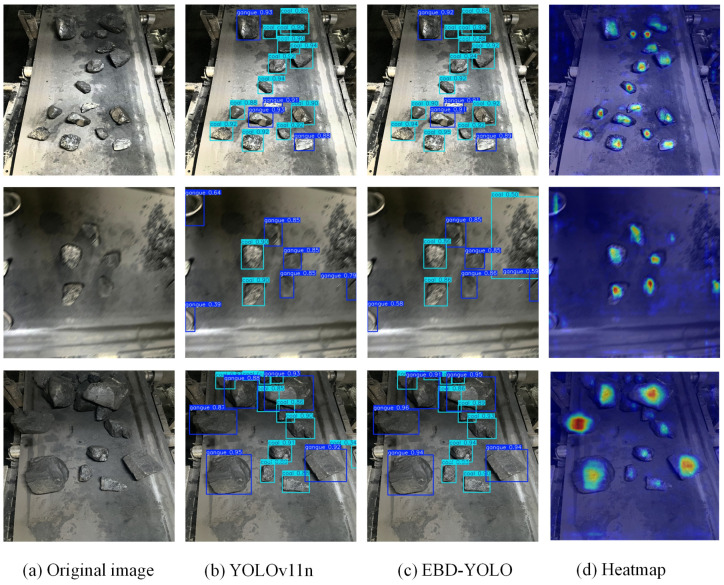
Experimental visualization results.

To assess the detection performance of the EBD-YOLO model, both the YOLOv11n model and the EBD-YOLO model were utilized to analyze images from the test set. The visualization results for coal gangue are displayed in Figure 8.

The prediction box for middling coal is represented in light blue and labeled as “coal”, while the prediction box for gangue is depicted in dark blue and labeled as “gangue”. As illustrated in Figure 8, both the YOLOv11n model and the EBD-YOLO model demonstrate effective capabilities in detecting both coal and gangue. The YOLOv11n model exhibits specific errors and omissions in detecting coal and gangue. In contrast, the EBD-YOLO model shows significant improvements in addressing these issues during the detection process. Furthermore, the detection accuracy of the EBD-YOLO model has markedly improved compared to the benchmark model, YOLOv11n.

The heatmap provides a clear and intuitive representation of the critical information emphasized by the model during the coal gangue detection process. This article utilizes the Grad-CAM [27] heatmap visualization strategy for analysis. Grad-CAM effectively captures gradient information from the final convolutional layer, enabling the prediction of weight information across various channels. Through a weighted summation process, a heatmap is generated and subsequently overlaid onto the original image. This representation illustrates the relative focus of attention during the detection process. Figure 8d presents the results of the thermodynamic diagram for the EBD-YOLO model. As shown in Figure 8d, the EBD-YOLO model exhibits a greater focus on the surface irregularities of coal and gangue during the detection process. This observation indicates that the enhanced model is more proficient at extracting relevant information during feature extraction, thereby confirming its robustness in complex scenarios.

## 4. Discussion

The EBD-YOLO model proposed in this study demonstrates promising results in the task of coal gangue detection and has made notable advancements. While the model performed well on this dataset, several limitations remain that warrant further improvement in future research, including:

(1) The model’s ability to generalize is constrained. The dataset used in the current model is derived exclusively from a single coal mine, which restricts its capacity to comprehensively represent the detection of coal gangue across diverse environments and equipment. Therefore, there is considerable potential for enhancing the model’s generalization capabilities. Future research should concentrate on improving the model’s adaptability across various conditions by incorporating a broader range of diverse datasets and training it under different scenarios.
(2) Although EBD-YOLO has shown advancements in detecting small targets, it still faces challenges with missed detections in specific scenarios. This issue is especially pronounced when identifying overlapping targets or extremely small objects. To address this issue, future research should concentrate on enhancing the robustness and small target detection capabilities of the model in complex environments. This can be accomplished by further optimizing data augmentation techniques and exploring additional improvement modules, such as the incorporation of multi-scale feature extraction and advanced attention mechanisms.

## 5. Conclusions

(1) This article presents an enhanced coal gangue detection model, EBD-YOLO, which is based on the improved YOLOv11n framework. First, the EMA attention mechanism is integrated into the C3k2 module of the backbone network to enhance the model’s ability to extract surface features of coal gangue while maintaining its complexity. Second, the BiFPN module has been incorporated to replace the feature fusion component of the original model, thereby improving the model’s capability to detect coal gangue across various scales. Finally, within the head network, the DyHead detection head is introduced to enhance both the detection accuracy and robustness of the model by integrating scale, spatial, and task-specific attention mechanisms.
(2) The heat map visualization indicates that the enhanced EBD-YOLO model places greater emphasis on the intrinsic characteristics of coal gangue compared to the original YOLOv11n model.
(3) The experimental results indicate that the EBD-YOLO model improves recognition speed while minimizing missed detections and false positives, all while maintaining a high level of detection accuracy. This performance meets the detection requirements for sorting scenarios in underground coal mining. In future research and initiatives, the model’s real-time performance and computational complexity will be further refined to enhance its subsequent deployment.

## Figures and Tables

**Table 1:** Ablation experiment.

Model	C3k2-EMA	BiFPN	DyHead	P	R	mAP @0.5	Params/M	FPS /f·s^−1^
baseline				85.3	80.2	87.8	2.58	82.6
①	√			84.1	82.0	88.5	2.61	80.6
②		√		86.8	78.2	89.0	1.92	90.9
③			√	83.0	84.5	88.1	3.10	84.2
④	√	√		83.5	83.4	89.3	2.56	82.47
⑤	√		√	88.5	84.6	91.1	3.14	86.95
⑥		√	√	87.6	82.5	89.3	2.60	89.38
⑦	√	√	√	88.7	83.9	91.7	2.60	90.91

Note: √ indicates the introduction of this module.

**Table 2:** Comparison experiment table of different models.

Model	P	R	mAP@0.5	Params/M	FLOPS/G	FPS/f·s^−1^
YOLOv3 -tiny	84.7	78.9	86.5	8.67	12.9	95.24
YOLOv5s	88.5	80.8	89.8	7.02	15.8	94.34
YOLOv8n	88.1	80.8	88.6	2.68	6.8	100.00
YOLOv10n	86.8	80.5	87.0	2.70	8.2	108.70
YOLOv11s	88.1	82.3	90.8	9.41	21.3	75.75
YOLOv11n	85.3	80.2	87.8	2.58	6.3	82.6
EBD-YOLO	88.7	83.9	91.7	2.60	7.9	90.91

**Table 3:** Performance comparison of YOLOv11n with different C3K2 modules.

Model	mAP@0.5	Params/M	FLOPS/G	FPS/f·s^−1^
YOLOv11n	87.8	2.58	6.3	82.6
+C3k2-EMA	88.5	2.61	6.6	80.6
+C3k2-CA	87.6	2.63	6.8	83.5
+C3k2-ECA	88.1	2.59	6.5	82.5
+C3k2-Faster	87.0	2.29	5.8	84.3
YOLOv11n	87.8	2.58	6.3	82.6

**Table 4:** Comparative experiment of different coal gangue models.

Model	mAP@0.5	Params/M	FLOPS/G	FPS/f·s^−1^
EBD-YOLO	91.7	2.60	7.9	90.91
HGTC-YOLOv8n [17]	87.6	2.64	8.0	75.55
SS-YOLOv3-tiny [19]	90.5	10.61	16.3	46.85
SG-YOLO [20]	87.8	3.68	8.0	80.21

## Data Availability

All data generated or analyzed during this study are included in this published article. The original contributions presented in the study are included in the article, further inquiries can be directed to the corresponding author.
